# Impulsivity and reward and punishment sensitivity among patients admitted to a specialized inpatient eating disorder treatment program

**DOI:** 10.3389/fpsyt.2024.1325252

**Published:** 2024-05-20

**Authors:** Mary K. Martinelli, Colleen C. Schreyer, Irina A. Vanzhula, Angela S. Guarda

**Affiliations:** Department of Psychiatry and Behavioral Sciences, Johns Hopkins University School of Medicine, Baltimore, MD, United States

**Keywords:** eating disorders, inpatient treatment, treatment, impulsivity, reward sensitivity, punishment sensitivity, personality

## Abstract

**Introduction:**

Eating disorders (EDs) are conceptualized as disorders of under- and over-control, with impulsivity reflecting under-control. Extant research indicates that impulsivity and related factors such as reward sensitivity and punishment sensitivity may serve as trait-level transdiagnostic risk and/or maintenance factors in EDs. Findings on impulsivity and reward and punishment sensitivity by diagnosis are mixed and research on the relationship between these factors and ED symptoms, hospital course, and treatment outcomes is limited.

**Methods:**

Participants (*N* = 228) were patients admitted to a specialized inpatient behavioral treatment program for EDs who agreed to participate in a longitudinal study and completed self-report measures of impulsivity, reward sensitivity, and punishment sensitivity at admission. Weight and ED symptomatology were measured at admission and discharge. Hospital course variables included length of stay and premature treatment dropout.

**Results:**

Impulsivity was lower in individuals with anorexia nervosa (AN) restricting type compared to those with AN binge/purge type or bulimia nervosa; no other group differences were observed. Higher impulsivity was associated with greater bulimic symptoms on the Eating Disorder Inventory 2 (EDI-2) at admission. Impulsivity was not related to ED symptoms, weight outcomes, length of hospital stay, or treatment dropout at program discharge.

**Conclusion:**

Impulsivity may help distinguish restrictive versus binge/purge EDs, but does not necessarily relate to discharge outcomes in an intensive inpatient ED program. Findings from this study provide novel contributions to the literature on personality traits in EDs and have important clinical implications. Results suggest that patients with higher levels of impulsivity or reward and punishment sensitivity can be expected to respond to inpatient treatment. Suggestions for future research are discussed.

## 1 Introduction

Prior research has focused on trying to better understand individual-level variability in clinical course and treatment response within eating disorder (ED) diagnostic categories to improve treatment outcomes (1, 2). However, the current categorical diagnostic system proposed in The Diagnostic and Statistical Manual of Mental Disorders Fifth Edition (DSM-5) (3) has considerable limitations including poor diagnostic stability over time and heterogeneous symptom profiles within diagnosis (4, 5). As such, some have recommended classifying individuals with EDs based on comorbid psychopathology and associated features, such as personality traits (6). One such trait is impulsivity, which has been identified as a potential transdiagnostic risk and maintenance factor for EDs. Broadly defined as a tendency to act without forethought or planning and without adequate regard for potential risks or negative consequences (7, 8), impulsivity is understood as a multi-dimensional construct encompassing a number of lower-order behavioral, cognitive, and emotional facets (9, 10). Traditionally, bulimia nervosa (BN) has been viewed as a disorder of impulsivity, while anorexia nervosa (AN) has been viewed as a disorder of compulsivity, however, more recent theoretical models conceptualize impulsivity and compulsivity in EDs as existing on separate continuums that may overlap within the same individual (11, 12).

Related to impulsivity, Reinforcement Sensitivity Theory (RST; 13) describes two motivational factors thought to underlie impulsivity: the behavioral inhibition system (BIS, i.e., punishment sensitivity) involved in inhibiting behaviors or avoiding situations that may result in negative consequences, and the behavioral activation system (BAS, i.e., reward sensitivity), involved in motivating approach behavior toward rewarding stimuli. Like the related construct of impulsivity, reward sensitivity and punishment sensitivity are personality traits that may influence risk and maintenance of EDs. For instance, many ED symptoms (binge eating, dietary restriction) can be motivated by approaching rewarding stimuli (e.g., food) or avoiding punishment (e.g., weight gain, physical discomfort after eating, distress about eating feared or unfamiliar foods). Transdiagnostically, individuals with EDs consistently endorse higher punishment sensitivity compared to healthy controls (14–19) and individuals with restrictive EDs tend to endorse lower reward sensitivity (14, 15, 17) although the latter finding is less consistent across studies.

Trait impulsivity and BIS/BAS may help to explain heterogeneity in symptom profiles across ED diagnoses. For example, despite their overlapping diagnostic underpinnings, inter-individual differences in impulsivity and BIS/BAS may explain why individuals with BN and AN binge/purge subtype (AN-BP) are more prone to bingeing and purging as a consequence of dietary restriction or in response to negative emotions while those with AN restricting subtype (AN-R) are able to overcome their biological urge to eat in order to engage in severe calorie restriction. In support of this hypothesis, a number of studies have found higher impulsivity among those with binge/purge EDs compared to those with AN-R (14, 20–22), and impulsivity tends to be significantly related to measures of bulimic symptoms (23–25). Studies have also found higher punishment sensitivity (BIS) and lower reward sensitivity (BAS) in AN-R compared to AN-BP and BN (14, 15, 26, 27). Others however, found no differences between diagnostic groups on impulsivity (14, 28–31), or on measures of reward sensitivity (16, 26, 27, 32, 33) or punishment sensitivity (15, 17, 26, 32). One explanation for these discrepancies across studies is that personality traits may be more robustly related to ED symptoms rather than diagnostic categories (34, 35). Given that prior studies primarily assessed differences in impulsivity and BIS/BAS by diagnosis, research that examines whether these traits relate to ED symptomatology within individuals with EDs is warranted. Discrepancies in the literature may also reflect the complex, multifaceted nature of impulsivity and reward sensitivity whereby different facets of these traits may relate to some ED symptoms more than others. For instance, Claes et al. (14) examined three dimensions of impulsivity using the Barratt Impulsiveness Scale (36) and found that while individuals with BN and AN-BP had higher motor and non-planning impulsivity than individuals with AN-R, groups did not differ on attentional impulsivity. Further research in mixed ED samples is needed to clarify the relationship between multidimensional personality traits and ED symptomatology, especially in higher acuity samples such as inpatients with EDs. Prior studies have primarily focused on comparing AN and BN, therefore, research that includes other ED diagnoses such as avoidant/restrictive food intake disorder (ARFID) and other specified feeding or eating disorder (OSFED) would add to this literature.

Individual differences in trait impulsivity and BIS/BAS may also influence ED treatment course and outcomes. Higher impulsivity has been associated with poorer response to treatment (23, 37) and greater likelihood of treatment dropout (38, 39), whereas others have found that baseline impulsivity and BIS/BAS did not predict treatment course or outcomes (31, 40). The majority of these outcome studies were conducted in outpatient treatment samples (23, 31, 39) or mixed inpatient/outpatient samples (38). Only two studies examined inpatient ED treatment outcomes, and one had a relatively small sample size (40) while the other study only included individuals with AN (37). The extent to which impulsivity is related to course and outcomes in specialized inpatient ED treatment is important, as these findings may differ from outpatient treatment and may inform individualized treatment. For instance, it is possible that the controlled setting of inpatient treatment may provide the structure and motivation necessary to help those with high impulsivity and BIS/BAS maintain focus and establish healthier eating and weight-related behaviors. Alternatively, the intensive refeeding and highly structured behavioral protocol typical of inpatient behavioral treatment can be highly distressing to patients who wish to maintain their current behaviors or low weight and thus may lead patients high in impulsivity or punishment sensitivity to drop out of treatment impulsively and prematurely. Those with higher reward sensitivity may also struggle with compliance while on the unit, for instance by attempting to surreptitiously engage in binge eating or excessive exercise. To improve suboptimal outcomes and retention rates in inpatient ED treatment, it may be necessary to look beyond known prognostic factors and explore novel transdiagnostic trait-level predictors of treatment outcomes that could inform the development of adaptations to existing treatments designed to better target trait-level risk and maintenance factors. Behavioral contingency management is integral to most inpatient treatment protocols (41) and findings from the addiction literature suggests that manipulating either the timing or amount of a positive or negative contingency may have differential effects on individuals depending on trait impulsivity (42–44). For example, manipulating access to exercise, food choice, or off-unit privileges may influence outcomes differentially depending on impulsivity or BIS/BAS traits.

The current study builds on existing literature and examines how trait impulsivity, reward sensitivity, and punishment sensitivity relate to ED symptoms and treatment outcomes among patients admitted to a specialized inpatient ED program. Specifically, we aimed to (1) investigate how multidimensional trait impulsivity and BIS/BAS relate, cross-sectionally, to diagnosis, ED symptoms, and body weight at admission, and (2) examine how personality factors measured at admission relate longitudinally to hospital course, weight outcomes, and ED symptoms at program discharge. We hypothesized that greater impulsivity and reward sensitivity, and lower punishment sensitivity would be associated with higher body weight and greater bulimic symptoms at admission and discharge, shorter length of hospital stay, and greater likelihood of treatment dropout.

## 2 Materials and methods

### 2.1 Participants

Eligible participants were adults and adolescents admitted to a specialized behavioral integrated inpatient-partial hospitalization program for EDs between May 2012 – July 2022 who consented to participate in a longitudinal research study (*n* = 237) and were fluent English speakers. Participants were included if they completed self-report personality measures at admission (*n* = 232, (excluding five participants who did not complete the BIS-11 and/or BIS/BAS). Binge eating disorder was considered for inclusion but was ultimately excluded due to small sample size (*n* = 3). We also excluded one participant who was admitted directly to partial hospital without attending inpatient treatment. Thus, the final sample included 228 patients diagnosed with AN-R, AN-BP, BN, OSFED, or ARFID. ED diagnoses were determined by the Structured Clinical Interview for DSM-5 research version (SCID-5-RV; 45).

### 2.2 Procedure

These data were collected as part of an ongoing, longitudinal study of response to intensive treatment in patients diagnosed with EDs. The study was approved by the Institutional Review Board of the Johns Hopkins University School of Medicine. Questionnaire data was collected using paper and pencil (online survey after March 2020) at two time points, within the first week of admission (T1) and within one week of program discharge (T2). Demographic and clinical course data abstracted from the electronic medical record included height, gowned morning weight at admission and discharge, target weight (for participants on weight gain protocol) length of stay, reason for discharge, and problematic behaviors observed by clinical staff during admission.

### 2.3 Treatment protocol

The inpatient eating disorders program employs a structured 100% meal-based behavioral treatment protocol delivered within a multidisciplinary integrated, inpatient program. Primary treatment goals include rapid weight restoration for underweight patients and normalization of eating and weight control behaviors. See Guarda et al. (46) for additional description of the treatment program.

### 2.4 Measures

#### 2.4.1 Structured clinician interview for DSM-5

The ED section of SCID-5-RV was used to determine participant diagnoses (45). The interviews were administered at admission by postdoctoral fellows or research assistants supervised by a licensed clinical psychologist. Participants admitted prior to 2015 were evaluated using the SCID-IV, and diagnoses were later re-assessed using DSM-5 criteria. The eating disorders module of the SCID-5-RV was administered by only one rater, therefore inter-rater reliability is not available.

#### 2.4.2 Weight variables

Height and weight in a gown measured at program admission and discharge were used to calculate BMI at each timepoint. Individual target weight was set as a four-pound range (1.8 kilograms) based on the patient’s age, sex, and height centered on a BMI of 20.5 kg/m^2^ for patients over age 25. For patients aged 16–24, target weight was adjusted by subtracting one pound (0.45 kilogram) per year of age below 25. For adolescents <16 years, target weight was set using growth charts or minimally by the 25^th^ BMI percentile when these were not available. Participants admitted below target weight were placed on a previously described weight gain nutritional protocol (47). Percentage of ideal body weight (IBW) at admission and discharge was computed by dividing current weight by target weight and multiplying by 100.

#### 2.4.3 Personality measures

##### 2.4.3.1 Barratt Impulsiveness Scale

The BIS-11 (36) is a 30-item self-report measure of trait impulsivity. Total BIS-11 impulsivity score and three subscale scores were measured in this study: attentional impulsivity (inability to focus or concentrate), motor impulsivity (acting without thinking), and non-planning impulsiveness (lacking forethought). Items are rated on a scale from 1 (almost never) to 4 (almost always). Total score ranges from 30 to 120 with scores ≥72 thought to capture those with high impulsivity, scores 52-71 considered normal and scores <52 considered low (i.e., over-controlled). The BIS-11 has good reliability and criterion validity (48). In the current study, the BIS-11 total score demonstrated good internal consistency (Cronbach’s α = .86) and BIS-11 subscales demonstrated acceptable internal consistency (Cronbach’s alphas: Attentional, α = 0.78; Motor, α = 0.69; Non-Planning, α = 0.76).

##### 2.4.3.2 Behavioral Inhibition and Behavioral Activation Scale

The BIS/BAS (49) is a 24-item scale designed to measure two general motivational systems that underlie behavior: a behavioral approach system (BAS; reward sensitivity or motivation to move towards something desired) and behavioral inhibition system (BIS; punishment sensitivity or motivation to move away from something unpleasant). There is one BIS-related scale and there are three BAS-related subscales: BAS-drive (motivation to pursue one’s desires or goals), BAS-fun seeking (motivation to seek out and spontaneously approach rewarding experiences in the environment), and BAS-reward responsiveness (anticipation and positive response to reward). The BIS/BAS has good psychometric properties (49). For the current study, internal consistencies measured via Cronbach’s alpha were acceptable (BAS drive: α = 0.75, BAS reward responsiveness: α = 0.74, BAS fun seeking: α = 0.69, BIS: α = 0.76).

#### 2.4.4 Measures of ED symptoms

##### 2.4.4.1 Eating Disorder Examination Questionnaire 6.0

The EDE-Q 6.0 assesses the frequency of disordered eating behaviors over the past 28 days and includes four subscales: restraint, eating concern, shape concern, and weight concern (50). The restraint subscale was not administered at discharge. The EDE-Q 6.0 has demonstrated good reliability and validity (51, 52). Individual item data for the EDE-Q 6.0 was not available to compute internal consistency.

##### 2.4.4.2 The Eating Disorder Inventory-2

The EDI-2 (53) is a 91-item self-report questionnaire designed to measure psychological features and behavioral traits commonly associated with AN and BN. The EDI-II has 11 subscales; three were measured in the current study: Drive for Thinness, Bulimia, and Body Dissatisfaction. The EDI-2 has demonstrated good reliability and validity in sample of individuals with EDs (54). In the current study, internal consistency ranged from good to excellent (Cronbach’s alphas: Drive for Thinness, α = 0.93; Bulimia, α = 0.90; Body Dissatisfaction, α = 0.92).

#### 2.4.5 Measures of comorbid psychopathology

##### 2.4.5.1 The State-Trait Anxiety Inventory

The STAI (55) is a 40-item self-report scale that measures anxiety as experienced in the moment (state subscale) and as a stable personality trait (trait subscale). Only the trait subscale was used in the current study. The STAI has demonstrated good reliability and validity (56, 57). In the current study, the STAI trait subscale (STAI-T) demonstrated excellent internal consistency (Cronbach’s α = 0.94).

##### 2.4.5.2 Beck Depression Inventory-II

The BDI-II (58) is a 21-item self-report measure that assesses depression symptoms in both adults and adolescents. The BDI-II has strong psychometric properties, including internal consistency and factor validity (58, 59). In the current study, internal consistency was excellent (Cronbach’s α = 0.91).

#### 2.4.6 Hospital course

Length of stay was calculated by subtracting admission from discharge date. Treatment dropout was coded as a dichotomous variable (yes/no) to indicate whether the participant prematurely dropped out of the program. Participants were coded as dropouts if they were discharged for reasons other than clinical improvement (e.g., non-compliance, elopement, patient-initiated for financial or other reasons). Problematic behaviors during treatment were determined via chart review for each participant. Dichotomous variables (yes or no) were created for eight problematic behaviors (i.e., restricting with weight loss, over-exercising, bingeing, vomiting, laxative or diuretic abuse, drug or alcohol abuse, suicidal ideation, and self-injurious behavior or suicide attempt). Variables were coded yes if a behavior was noted by the treatment team at any point during the participant’s hospitalization. A dichotomous summary variable was computed by coding yes if a participant exhibited *any* of the eight problematic behaviors during their hospital stay or no if they did not exhibit problematic behaviors.

### 2.5 Data analysis

Data were analyzed using SPSS version 28. Preliminary analyses were conducted to determine availability of data and test for potential covariates. Approximately 63% of participants had available data on the EDE-Q at T2 and approximately 32% had data available on the EDI-2 at T2. Of note, the EDI-2 was collected at T2 (final program discharge) only among participants who stepped down to the integrated partial hospitalization program (*n* = 139, 61%). Of this group, approximately 52.5% had T2 EDI-2 data available. All other T2 variables (i.e., EDE-Q, discharge BMI, discharge %IBW) were measured at program discharge (i.e., at inpatient discharge for those attending inpatient only or at partial hospital discharge for those attending the integrated inpatient-partial hospitalization program). In preliminary analyses, independent samples *t*-tests were used to compare participants who attended the integrated inpatient-partial hospitalization program (*n* = 139, 61%) to those who attended inpatient only (*n* = 89, 39%). The two groups did not significantly differ on BIS-11 total or subscales, or on BIS/BAS subscales (all *p*’s >.05).

Age, depression, and anxiety were considered as potential covariates for study analyses. In preliminary bivariate correlations, BIS-11 total impulsivity was significantly correlated with age, depression, and anxiety (*p*’s <.05). For the BIS/BAS, bivariate correlations revealed that BAS-fun seeking, BAS-reward responsiveness, and the BIS subscale were significantly correlated with depression and anxiety (*p*’s <.05), but not age (*p* = .37, .89, .36, respectively), while BAS-drive was not associated with age, depression, or anxiety (*p* = .42, .99, .19, respectively). Subsequent regression analyses controlled for age, depression and anxiety in models with BIS-11, and controlled for depression and anxiety in models with BIS/BAS.

A one-way analysis of variance (ANOVA) was conducted to test for differences in personality factors (i.e., BIS-11 total and subscales, and BIS/BAS subscales) by DSM-5 ED diagnosis. Alpha level was set to *p* <.05 for ANOVA. *Post-hoc* pairwise comparisons were conducted with a Bonferroni correction for multiple comparisons.

Bivariate correlations were used to examine associations between personality measures at admission (BIS-11 and BIS/BAS) and all continuous variables of interest, including ED symptoms (EDE-Q and EDI-2) at admission and discharge, weight variables (BMI, percent IBW) at admission and discharge, and length of hospital stay. Given the high number of correlation tests conducted, a Bonferroni correction was applied to account for multiple tests (.05/8 personality variables) making the threshold for significance *p* <.00625. For any significant bivariate correlations, a series of follow-up hierarchical multiple regression analyses were conducted to assess the unique relationship between the measures controlling for relevant covariates such as age, anxiety, and depression. Three separate hierarchical regression models were conducted for each dependent variable (DV) of interest: i) a model with age, anxiety, and depression entered as covariates in step 1 followed by BIS-11 attentional, motor, and non-planning subscales entered in step 2, ii) a model with age, anxiety, and depression entered as covariates in step 1 followed by BIS-11 total score in step 2, and iii) a model with anxiety and depression entered as covariates in step 1 followed by BAS drive, BAS fun seeking, BAS reward responsiveness, and BIS subscale entered in step 2. For models with discharge BMI or length of hospital stay as the DV, admission BMI was added as a covariate in step 1. For models with discharge EDE-Q or EDI-2 as the DV, admission EDE-Q or EDI-2 was added as a covariate in step 1. *F*-change significance was evaluated at step 2 to determine whether the impulsivity variable(s) significantly predicted the DV above and beyond control variables. Coefficient values were used to determine significance of each predictor in the model controlling for other predictors. A Bonferroni correction was applied to account for multiple tests conducted on each DV of interest (.05/3 tests) making the threshold for significance *p* <.0167.

To assess the relationship between personality factors at admission and categorial discharge measures (i.e., treatment dropout, presence of problematic behaviors during admission), a series of binary logistic regression analyses were conducted with the categorical variable of interest entered as the DV and predictors entered to the model using the same steps described above. Chi-square statistics were examined at step 2 to determine if adding the personality variables significantly improved prediction of the categorical DV above and beyond covariates. Wald statistics were examined to determine whether an individual predictor significantly contributed to the prediction of the DV. A Bonferroni correction was applied to account for multiple tests conducted on each DV of interest (.05/3 tests) making the threshold for significance *p* <.0167.

## 3 Results

### 3.1 Sample characteristics

Descriptive statistics for demographic, clinical, and self-report data are presented in **Table 1**. As shown, the majority of the sample was female and on weight gain protocol at admission. Average age of the sample was 29.53 years. Drop-outs and completers did not differ on baseline sociodemographic characteristics (sex, age), ED symptoms (EDE-Q, EDI-2), or comorbid psychopathology (anxiety, depression). However, there were significant differences in the proportion of drop-outs by ED diagnosis (χ^2^(*4*) = 18.74, *p* <.001) with AN-R and AN-BP having a higher dropout rate (49-54%) compared to other ED diagnoses (21-33%). Accordingly, drop-outs also had significantly lower admission BMIs (*M* = 16.98, *SD* = 4.97) compared to completers (*M* = 18.87, *SD* = 4.97), *t* =2.80, *p* =.006.

**Table 1:** Demographic, clinical, and self-report variables (*N* = 228).

	Admission	Discharge
	*M* (SD) or *n* (%)	*M* (SD) or *n* (%)
Age (years)	29.53 (13.58)	
Female sex	206 (90.4%)	
On weight gain protocol	172 (75.4%)	
BMI (kg/m^2^)	18.12 (5.04)	21.04 (4.21)
Percent IBW^a^	79.99 (10.39)	96.40 (8.62)
Length of stay (days)		50.57 (28.52)
Primary diagnosis		
AN-R	74 (32.5%)	
AN-BP	55 (24.1%)	
BN	33 (14.5%)	
OSFED	39 (17.1%)	
ARFID	27 (11.8%)	
BDI-II Total	24.65 (12.79)	
STAI Trait anxiety	54.32 (13.97)	
BIS-11 Total	63.24 (12.66)	
Attentional	18.32 (4.80)	
Motor	20.89 (4.78)	
Non-Planning	24.04 (5.87)	
BIS/BAS		
BAS Drive	10.54 (2.77)	
BAS Fun Seeking	10.52 (2.68)	
BAS Reward Responsiveness	16.50 (2.66)	
BIS subscale	23.65 (3.63)	
EDE-Q		
Restraint^b^	2.90 (2.16)	
Eating Concern^b^	2.66 (1.72)	1.54 (1.38)^d^
Weight Concern^b^	3.24 (1.87)	2.51 (1.83)^e^
Shape Concern^c^	3.47 (1.94)	3.05 (1.98)^d^
EDI-2		
Drive for Thinness	28.21 (11.37)	25.14 (10.42)^f^
Bulimia	16.49 (9.05)	13.01 (5.91)^g^
Body Dissatisfaction	36.80 (12.31)	35.53 (12.84)^g^

BMI, body mass index; IBW, ideal body weight; AN-R, anorexia nervosa, restricting type; AN-BP, anorexia nervosa, binge/purge type; BN, bulimia nervosa; OSFED, other specified feeding/eating disorder; ARFID, avoidant/restrictive food intake disorder; BDI-II, Beck Depression Inventory; STAI, State-Trait Anxiety Inventory; BIS-11, Barratt Impulsiveness Scale; BAS, Behavioral Activation Scale; BIS, Behavioral Inhibition Scale; EDE-Q, Eating Disorder Examination Questionnaire; EDI-2, Eating Disorder Inventory.

^a^Weight gain protocol only (n = 172), ^b^n = 169, ^c^n = 168, ^d^n = 143, ^e^n = 142, ^f^n = 73, ^g^n = 72.

### 3.2 Differences in personality factors by DSM-5 ED diagnosis

A one-way ANOVA of ED diagnosis on BIS-11 and BIS/BAS revealed significant main effects of diagnosis for BIS-11 motor, non-planning, and total impulsivity and BAS fun seeking (**Table 2**). *Post-hoc* comparisons with a Bonferroni correction revealed that the AN-R group had lower levels of motor impulsivity than the AN-BP group (mean difference = -4.00, *p* <.001), lower non-planning impulsivity than the BN group (mean difference = -3.75, *p* =.02), and lower total impulsivity than AN-BP (mean difference = -8.77, *p* <.001) and BN (mean difference = -7.96, *p* =.02). AN-R had lower BAS fun seeking than AN-BP (mean difference = -1.46, *p* =.02). No differences were observed for ARFID or OSFED.

**Table 2:** One-way ANOVA comparing impulsivity scores by DSM-5 eating disorder diagnosis.

	AN-R *n* = 74		AN-BP *n* = 55		BN *n* = 33		OSFED *n* = 39		ARFID *n* = 27		*F*	*p*	Significant *post-hoc* tests^a^
	*M*	*SD*	*M*	*SD*	*M*	*SD*	*M*	*SD*	*M*	*SD*			
BIS-11													
AT	17.29	5.20	19.47	4.41	18.82	5.14	18.55	3.86	17.83	4.93	1.86	0.118	
MT	19.15	3.80	23.15	4.34	21.82	5.35	20.49	5.34	20.48	4.69	6.54	<.001***	AN-R < AN-BP
NP	22.80	6.02	25.38	5.67	26.55	5.57	23.21	5.58	22.85	5.48	3.70	0.006**	AN-R < BN
Total	59.23	12.55	68.00	12.03	67.19	12.99	62.25	11.62	61.16	11.48	5.21	<.001***	AN-R < AN-BP; AN-R < BN
BIS/BAS													
BAS-DR	10.19	2.87	11.38	2.63	10.61	2.77	10.41	2.78	9.93	2.50	1.96	0.101	
BAS-FS	9.76	2.69	11.22	2.63	10.52	2.86	10.67	2.56	11.00	2.30	2.77	0.028*	AN-R < AN-BP
BAS-RR	16.18	2.98	17.05	2.31	16.61	2.32	16.33	2.94	16.41	2.34	0.93	0.446	
BIS subscale	23.93	3.68	23.36	3.83	24.30	3.12	24.13	3.04	21.96	4.09	2.14	0.077	

BIS-11, Barratt Impulsiveness Scale; AT, Attentional; MT, Motor; NP, Non-Planning; BAS, Behavioral Activation Scale; DR, Drive; FS, Fun Seeking; RR, Reward Responsiveness; BIS, Behavioral Inhibition Scale; AN-R, anorexia nervosa, restricting type; AN-BP, anorexia nervosa, binge/purge type; BN, bulimia nervosa; OSFED, other specified feeding/eating disorder; ARFID, avoidant/restrictive food intake disorder.

^a^Significant in post-hoc tests with Bonferroni adjustment for multiple comparisons.

*p <.05, **p <.01, ***p <.001.

### 3.3 Cross-sectional relationship between personality factors and T1 weight and ED symptoms

Bivariate correlations between admission impulsivity measures and admission weight and ED measures are presented in **Table 3**. Admission BMI and percent IBW were not significantly correlated with any BIS-11 or BIS/BAS scales after adjusting for multiple comparisons (*p*’s >.00625). A number of significant correlations between personality measures and EDE-Q and EDI-2 scales were observed, with the exception of EDE-Q restraint, which was not significantly correlated with any BIS-11 or BIS/BAS dimensions. BIS-11 attentional impulsivity was the variable found to be most frequently related to ED symptoms: higher attentional impulsivity was associated with greater eating, weight, and shape concerns on the EDE-Q (*p*’s = .001, .003, <.001, respectively), as well as greater drive for thinness, bulimia symptoms, and body dissatisfaction on the EDI-2 (*p*’s <.001). The motor and non-planning impulsivity subscales were only significantly correlated with EDI-2 bulimia symptoms (*p*’s <.001). Higher BIS-11 total score was also correlated with greater bulimia symptoms (*p* <.001) and was additionally related to greater body dissatisfaction on the EDI-2 (*p* = .001), and greater shape concern on the EDE-Q (*p* = .004). Greater punishment sensitivity as measured by the BIS subscale of the BIS/BAS was positively associated with EDE-Q eating, weight, and shape concerns (*p*’s = .004, .001, <.001, respectively) and EDI-2 body dissatisfaction and drive for thinness (*p*’s <.001), while reward sensitivity (BAS subscales) was not related to any ED symptoms.

**Table 3:** Bivariate correlations between impulsivity, weight, and ED symptoms at program admission.

T1 variables	BIS-11 AT	BIS-11 MT	BIS-11 NP	BIS-11 Total	BAS DR	BAS FS	BAS RR	BIS Subscale
BMI	.079	.111	.148*	.141*	-.074	-.011	-.103	.034
%IBW^a^	.074	.065	.116	.106	-.078	.043	-.065	-.043
EDE-Q Restraint^b^	.171*	.016	.004	.070	.060	-.126	-.046	.187*
EDE-Q Eating concern^b^	.242*	.115	.149	.200*	.046	-.058	.025	.222*
EDE-Q Weight concern^b^	.229*	.058	.160*	.179*	-.033	-.155*	-.088	.255*
EDE-Q Shape concern^c^	.266*	.096	.192*	.221*	.021	-.131	-.075	.303*
EDI-2 Drive for thinness	.239*	.045	.123	.165*	.037	-.103	-.030	.319*
EDI-2 Bulimia	.233*	.260*	.333*	.341*	.143*	.136*	.117	.085
EDI-2 Body dissatisfaction	.280*	.084	.170*	.217*	-.093	-.160*	-.127	.300*

N = 228 unless otherwise specified; Results with asterisk (*) are significant based on unadjusted p <.05; Bolded results with asterisk (bold, *) are significant based on p <.00625 (adjustment for multiple tests); T1, time 1 (admission); ED, eating disorder; BIS-11, Barratt Impulsiveness Scale; AT, Attentional; MT, Motor; NP, Non-Planning; BAS, Behavioral Activation Scale; DR, Drive; FS, Fun Seeking; RR, Reward Responsiveness; BIS, Behavioral Inhibition Scale; BMI, body mass index (kg/m^2^); %IBW, percent ideal body weight; EDE-Q, Eating Disorder Examination Questionnaire; EDI-2, Eating Disorder Inventory.

^a^Weight gain protocol only (n = 172).

^b^N = 169.

^c^N = 168.

Follow-up hierarchical regressions were conducted for significant bivariate correlations to evaluate whether the relationships remain significant after controlling for relevant covariates. Significance was determined using a Bonferroni corrected alpha level of .0167. **Table 4** shows results from a series of hierarchical regressions with T1 EDI-2 bulimia symptoms as DV. In Model 1, BIS-11 attentional, motor, and non-planning subscales were added as predictors to a regression model containing age, anxiety, and depression. The overall model was significant (*F* = 5.27, *p* <.001) and adding the impulsivity subscales significantly improved prediction of bulimia symptoms above and beyond control variables (*F*-change = 7.85, *p* <.001). However, only the non-planning subscale emerged as a significant predictor (*p* = .006). In Model 2, higher BIS-11 total score was significantly associated with greater bulimia symptoms at admission controlling for age, depression, and anxiety (*p* <.001). The BIS/BAS scales were not significant predictors of EDI-2 bulimia symptoms controlling for depression and anxiety (Model 3).

**Table 4:** Model summary and coefficients of hierarchical regression for EDI-2 bulimia at admission (*N* = 228).

Model	Step^a^	Predictors	*B*	*β*	*t*	*R* ^2^	*F-*change	*F*
	1	Age	0.01	0.02	0.26	0.03	2.46	2.46
		Depression	0.02	0.03	0.28			
		Anxiety	0.10	0.16	1.60			
1	2	BIS-11 Attentional	0.05	0.03	0.30	0.13	7.85*	5.27*
		BIS-11 Motor	0.20	0.11	1.31			
		BIS-11 Non-Planning	0.37	0.24	2.78*			
2	2	BIS-11 Total	0.23	0.33	4.67*	0.12	21.81*	7.47*
	1	Depression	0.02	0.03	0.30	0.03	3.68*	3.68*
		Anxiety	0.10	0.16	1.59			
3	2	BAS Drive	0.26	0.08	1.09	0.08	2.91*	3.21*
		BAS Fun Seeking	0.42	0.12	1.61			
		BAS Reward	0.30	0.09	1.07			
		BIS subscale	-0.11	-0.05	-0.57			

Results with asterisk (*) are significant based on unadjusted p <.05; Bolded results with asterisk (bold, *) are significant based on p <.0167 (adjustment for multiple tests); T1, time 1 (inpatient admission); EDI-2, Eating Disorder Inventory 2; BIS-11, Barratt Impulsiveness Scale; BAS, Behavioral Activation Scale; BIS, Behavioral Inhibition Scale; Depression, Beck Depression Inventory (BDI-II) total score; Anxiety, State-Trait Anxiety Inventory Trait subscale (STAI-T).

^a^Only variables unique to each step are presented.

Follow-up regressions were also conducted with EDE-Q eating, shape, and weight concerns as DVs, and with EDI-2 drive for thinness and body dissatisfaction as DVs, but no significant findings were observed (all *p*’s >.0167) (**Supplementary Tables 1, 2**).

### 3.4 Longitudinal relationship between personality measures and T2 weight and hospital course outcomes

Bivariate correlations between personality measures at admission with and discharge BMI, discharge percent IBW, and length of hospital stay are shown in **Table 5**. After correcting for multiple comparisons, impulsivity and reward/punishment sensitivity measures were not significantly correlated with BMI, percent IBW, or length of hospital stay at program discharge (*p*’s >.00625).

**Table 5:** Bivariate correlations between T1 personality factors and T2 weight outcomes, length of hospital stay, and ED symptoms.

T2 variables	*N*	BIS-11 AT	BIS-11 MT	BIS-11 NP	BIS-11 Total	BAS DR	BAS FS	BAS RR	BIS Subscale
Discharge BMI	228	.069	.099	.137*	.127	-.048	-.027	-.088	.017
Discharge %IBW^a^	172	.108	.088	.114	.126	-.009	.053	.018	-.057
Length of stay (days)	228	.128	-.047	.072	.064	-.025	-.136*	-.065	.128
EDE-Q Eating concern	143	.175*	.050	.059	.111	-.118	-.195*	-.074	.262*
EDE-Q Weight concern	142	.153	.047	.123	.132	-.021	-.152	-.094	.267*
EDE-Q Shape concern	143	.127	-.012	.100	.089	-.050	-.154	-.096	.316*
EDI-2 Drive for thinness	73	.272*	.134	.080	.191	-.108	-.132	-.214	.088
EDI-2 Bulimia	72	.257*	.302*	.314*	.362*	-.129	.105	-.238*	-.029
EDI-2 Body dissatisfaction	72	.212	.010	.014	.088	-.089	-.234*	-.135	.211

Results with asterisk (*) are significant based on unadjusted p <.05; Bolded results with asterisk (bold, *) are significant based on p <.00625 (adjustment for multiple comparisons); T1, time 1 (admission); T2, time 2 (discharge); ED, eating disorder; BIS-11, Barratt Impulsiveness Scale; AT, Attentional; MT, Motor; NP, Non-Planning; BAS, Behavioral Activation Scale; DR, Drive; FS, Fun Seeking; RR, Reward Responsiveness; BIS, Behavioral Inhibition Scale; BMI, body mass index (kg/m^2^); %IBW, percent ideal body weight; EDE-Q, Eating Disorder Examination Questionnaire; EDI-2, Eating Disorder Inventory 2.

^a^Weight gain protocol only.

Regarding dropout, the majority of participants were discharged from the program for clinical improvement (*n* = 138, 61%). Approximately 39% (*n* = 90) were classified as early dropouts. In a series of binary logistic regressions, impulsivity and reward/punishment sensitivity measures at admission were not significantly associated with likelihood of premature treatment dropout controlling for relevant covariates and admission BMI (*p*’s >.0167) (**Supplementary Table 3**). Regarding problematic behaviors (e.g., over-exercise, substance use, self-harm), 32% of participants (*n* = 73) exhibited one or more problematic behaviors during admission. In a series of binary logistic regressions, impulsivity and reward/punishment sensitivity measures did not significantly predict likelihood of engaging in problematic behavior during admission controlling for relevant covariates (*p*’s >.0167) (**Supplementary Table 4**).

### 3.5 Longitudinal relationship between personality measures and T2 ED symptoms

Bivariate correlations between BIS-11 and BIS/BAS and T2 ED symptoms are shown in **Table 5**. BIS-11 attentional, motor, and non-planning subscales were not significantly correlated with any ED symptoms at T2. Higher BIS-11 total at admission was only related to greater EDI-2 bulimia symptoms at discharge (*p* = .002). The BAS drive, fun seeking, and reward responsiveness subscales were not significantly associated with any EDI-2 or EDE-Q measures at T2. The BIS subscale of the BIS/BAS was significantly positively correlated with EDE-Q eating, weight, and shape concerns at T2 (*p*’s = .002, .001, <.001, respectively).

In a follow-up hierarchical regression BIS-11 total did not significantly predict T2 bulimia symptoms (*p* =.0168) after controlling for age, depression, anxiety, and T1 bulimia symptoms (**Supplementary Table 5**). Similarly, the BIS subscale of the BIS/BAS did not significantly predict eating, weight, or shape concerns at T2 (*p*’s = .591, .496, .836, respectively) in follow-up hierarchical regressions controlling for T1 ED symptoms and other relevant covariates (**Supplementary Table 6**).

## 4 Discussion

The purpose of this study was to investigate how trait-level factors of impulsivity, reward sensitivity, and punishment sensitivity relate cross-sectionally and longitudinally to ED symptoms, weight, and hospital course among patients admitted to a specialized inpatient behavioral treatment program for EDs. Findings from this study largely support the notion of higher impulsivity in those with binge/purge EDs compared to those with AN-R although findings also suggest that the relationship between bulimic symptomatology and impulsivity may be confounded by comorbid anxiety and depression symptomatology. Importantly, however, contrary to hypotheses we found that these personality factors do not appear to interfere with treatment adherence or discharge outcomes from inpatient ED treatment. This study provides novel contributions to the literature on personality factors and EDs. The study included a large inpatient treatment sample with a wider range of diagnoses than previous studies, explored how personality relates to clinical indices over multiple timepoints in treatment, and analyses controlled for potential confounding factors such as comorbid anxiety and depression symptomatology. Additionally, our use of multidimensional personality and ED symptom measures is in line with recommendations for investigating transdiagnostic, cross-cutting personality factors underlying EDs and comorbid psychopathology (11, 60), which could impact treatment outcomes.

### 4.1 Cross-sectional findings

#### 4.1.1 DSM-5 ED diagnosis

The finding of lower BIS-11 total, motor, and non-planning impulsivity, but not attentional impulsivity, in participants with AN-R compared to those with AN-BP or BN confirms findings from previous studies (14, 20, 21), as does the finding of lower BAS fun seeking among participants with AN-R compared to participants with AN-BP (14, 26). Lower levels of impulsivity and fun seeking in AN-R may help to explain how these individuals can overcome their biological urge to eat and engage in severe calorie restriction while those with AN-BP and BN are prone to bingeing and purging in response to strict dieting. We did not find significant differences between diagnostic groups on punishment sensitivity (BIS) in this study consistent with findings by Beck et al. (26) but in contrast to other studies reporting greater BIS in AN-R compared to AN-BP or BN (14, 61). One possible explanation for the discrepancy in findings is that the current study includes a wider range of ED diagnoses and therefore captures more variability in ED symptom profiles than prior studies. Additionally, given increasing recognition of limitations with categorical classification of EDs and the push for a shift to categorization based on shared psychopathology and comorbid personality traits, using diagnostic criteria may not be the most efficient method of understanding trait-level factors in EDs. No differences in impulsivity, reward sensitivity, or punishment sensitivity emerged between ARFID or OSFED and other ED diagnostic groups. Our inclusion of ARFID is particularly novel. No study to our knowledge has compared ARFID to other ED diagnoses on self-reported trait impulsivity and reward and punishment sensitivity. This is important given that ARFID is the newest ED diagnosis in DSM-5 and much about its shared and distinct etiological underpinning across EDs remains unknown. In contrast to the current study findings, one prior study comparing outpatients with AN and ARFID on a behavioral impulsivity task found greater reward-related impulsivity among ARFID compared to AN (62). The mixed findings may be due to differences in behavioral versus self-report measures or perhaps inpatients with ARFID are more similar to individuals with AN on measures of impulsivity. Alternatively, the small sample size of ARFID in the current study may have made it difficult to detect diagnostic differences. Thus, future research is needed to clarify the role of impulsivity as a potential transdiagnostic versus disorder-specific factor in ARFID and AN.

#### 4.1.2 Weight measures at admission

Impulsivity, reward sensitivity, and punishment sensitivity were not related to admission BMI or percentage below target body weight at admission. These findings are similar to prior research by Harrison et al. (31) which showed that BIS/BAS dimensions were not related to BMI at the start of outpatient treatment for AN. Although prior studies using behavioral measures of impulsivity indicate a positive relationship between impulsivity and higher weight at the start of ED treatment (63), our results and results from Harrison et al. (31) seem to suggest that this may not be true for self-report measures of trait impulsivity. Alternatively, differences between studies may be related to differences in BMI between samples given that the samples in the current study and the study by Harrison et al. (31) both had a mean BMI of approximately 18 kg/m^2^, whereas the mean BMI in Todisco et al. (63) was approximately 21 kg/m^2^.

#### 4.1.3 ED symptoms at admission

Impulsivity was significantly related to several eating disorder symptom scales on the EDE-Q and EDI-2 at admission. Most notably, greater EDI-2 bulimia symptoms at admission was related to higher BIS-11 total impulsivity and attentional, motor, and non-planning impulsivity subscales, however only total and non-planning impulsivity were significant after accounting for age, anxiety, and depression. These findings are consistent with prior research among individuals with BN showing a relationship between more frequent vomiting and poor long-term planning abilities (24). Given that non-planning impulsivity reflects a lack of forethought or future orientation, these findings suggest that individuals high in this dimension of impulsivity may be more likely to act on sudden urges to binge and purge without considering the associated dysphoria and negative physical consequences of these behaviors. The finding that higher punishment sensitivity (BIS) was correlated with greater eating, shape, and weight concerns, and greater drive for thinness and body dissatisfaction suggests that those with a greater tendency for anxiety-driven responding (i.e., avoidance) may be more likely to engage in disordered eating as a means to reduce fears of weight gain and low body dissatisfaction (64), however it is important to note that these findings were no longer significant after controlling for depression, and anxiety. As such, this tendency may be attributable to the high comorbidity rates between EDs and anxiety (65). It is interesting that reward sensitivity (BAS) was not significantly associated with ED symptoms in the current study. These findings are inconsistent with prior research showing a positive relationship between BAS reward responsiveness and global EDE-Q score at outpatient treatment admission (31). Differences between measures of reward and punishment sensitivity may explain inconsistencies in findings across studies. For example, another measure called the Sensitivity to Punishment and Sensitivity to Reward Questionnaire (SPSRQ) may show significant relationships to EDs due to the focus on social situations and social rewards, as opposed to more general reward sensitivity measured by the BIS/BAS (34). Future research should continue to clarify the nuances in reward sensitivity as it is related to EDs.

### 4.2 Longitudinal findings

#### 4.2.1 Weight outcomes and hospital course at discharge

In the current study, impulsivity and BIS/BAS at admission were not significantly related to BMI or percent target weight at program discharge. Prior studies were mixed regarding the relationship between weight outcomes and impulsivity, reward sensitivity, and punishment sensitivity during ED treatment. Harrison et al. (31) found that higher BAS reward responsiveness and BIS at the start of treatment predicted higher BMI at the end of treatment, whereas findings from Wildes et al. (37) did not support a longitudinal relationship between impulsivity and weight outcomes. Findings from Zerwas et al. (1) found that impulsivity was a positive prognostic predictor of weight recovery in AN. Perhaps the focus on rapid weight restoration or the structure of the behavioral protocol employed in the current study was powerful enough to result in similar weight restoration across participants regardless of admission personality trait levels. We also did not find a relationship between BIS-11 or BIS/BAS and length of hospital stay, consistent with some prior research (37) although this question has received less attention in the literature to date. Additionally, impulsivity and reward and punishment sensitivity at admission were not associated with likelihood of treatment dropout. These findings, although surprising in light of prior research showing that higher impulsivity is associated with greater likelihood of treatment dropout (38, 39), have important clinical implications as they suggest that even patients with higher impulsivity as reflected by the BIS-11 or by BIS/BAS scores are able to complete treatment with clinical improvement.

To our knowledge, this was the first study to examine the relationship between impulsivity and sensitivity to reward and punishment with problematic behaviors during hospitalization. Contrary to our hypotheses, impulsivity and reward and punishment sensitivity at admission did not predict likelihood of staff observed problematic behaviors during admission. One might assume that more impulsive patients would be more likely to engage in problematic behaviors (e.g., restriction, purging, substance use, self-harm) during admission given trait impulsivity has been posited as a potential shared risk and maintenance factor linking EDs and comorbid psychopathology (35). However, factors such as compulsivity and anxiety are also likely to contribute to behavioral dysregulation in patients with EDs, particularly when combined with negative affect or poor emotion regulation (12). It is possible that individuals across the impulsivity/compulsivity spectrum engaged in problematic behaviors at similar rates despite being influenced by different underlying emotional and behavioral processes. Alternatively, the behavioral protocol including contingency management and milieu therapy may have provided sufficient supervision and structure to help patients resist impulsive urges among those with higher trait impulsivity and BIS/BAS. Further, given that biological factors (e.g., malnutrition, vitamin deficiencies) have been shown to contribute to the manifestation of impulsivity in EDs (66), the emphasis on nutritional rehabilitation during inpatient treatment may help reduce impulsivity.

#### 4.2.2 ED self-report measures at discharge

Trait impulsivity, punishment sensitivity, and reward sensitivity at T1 did not predict ED symptoms on the EDE-Q or EDI-2 at T2. Of note, there were significant reductions in most of the EDE-Q and EDI-2 subscales from admission to discharge. Thus, findings suggest that patients can improve their ED symptoms during specialized inpatient treatment regardless of their pre-treatment levels of impulsivity. Few prior studies have included multidimensional self-report measures for both personality factors and ED symptoms. Nonetheless some evidence from studies in outpatient treatment samples found a link between higher sensitivity to punishment at baseline and greater EDE-Q global symptoms at the end of treatment (31) as well as between greater BIS-11 total impulsivity at admission and lower reduction in binge episodes at the end of treatment (23). Our findings contribute to the literature in this area by measuring multidimensional impulsivity and ED symptom measures longitudinally in a large mixed-diagnosis inpatient treatment sample.

### 4.3 Limitations

Several study limitations should be noted. Most analyses were correlational, thus we are unable to infer causal relationships between variables. Additionally, we did not have data on comorbid diagnoses (e.g., generalized anxiety disorder, major depressive disorder, borderline personality disorder), which would have been helpful given our interest in personality factors known to underlie EDs and comorbid psychopathology. Although our inclusion of participants with ARFID is novel and contributes to the literature on impulsivity in ARFID, our sample size for this group was small and may have limited the ability to detect differences across diagnoses. Specifically, although the ARFID group appeared to score similarly to AN-R on impulsivity and BIS/BAS, results of *post-hoc* comparisons did not reach statistical significance. Future studies with larger sample sizes are needed to improve overall generalizability and to confirm whether ARFID and AN-R share similar trait-level personality factors. ARFID participants in this inpatient treatment study may also present with lower BMIs compared to outpatient ARFID treatment samples. Our sample size for discharge outcomes was also relatively small which may have limited power to detect associations between impulsivity and discharge outcomes or may have introduced bias as those completing T2 questionnaires may have not been representative of the full T1 sample. Additionally, we included only self-report trait measures of impulsivity and sensitivity to punishment and reward. Although we were interested in examining the predictive validity of stable personality traits, results may not generalize to behavioral indices of impulsivity or sensitivity to reward and punishment (14, 67). Finally, we do not have information on longer term outcomes and relapse or recovery rates. Future research could consider including both self-report and behavioral measures within a longitudinal study of inpatient ED treatment that includes follow up within the high-risk period for relapse estimated as a year following weight restoration (68), as such research is currently lacking and may help clarify the role of stable vs state constructs as well as subjective vs objective measures of these factors.

### 4.4 Future directions

An improved understanding of the relationship between dimensions of impulsivity and reward sensitivity and ED symptoms in hospitalized patients with severe eating disorders is needed and may inform personalized approaches to care by elucidating the complex relationship between trait-level factors, symptoms and clinical severity. For instance, Liebman et al. (64) found that, among individuals high in punishment sensitivity, greater overvaluation of weight and shape was associated with greater frequency of non-compensatory purging behavior. Given our updated understanding of impulsivity and compulsivity as co-occurring dimensional traits rather than distinct extremes of a behavioral spectrum, such interactions are likely to occur within ED diagnoses and may shed light on mechanisms of ED thoughts and behavior. Given the role of emotional facets of impulsivity (e.g., positive and negative urgency) in EDs (69, 70), future research should explore distinctions between the impulsivity facets measured in this study (BIS-11) and emotion-focused measured of impulsivity. Lastly, in light of findings from this study, impulsivity may be more closely related to longer-term rather than short-term discharge outcome from intensive treatment. Some prior studies have reported a relationship between greater impulsivity and lower likelihood of remission at longitudinal follow-up (23, 40, 71). For example, although Testa et al. (40) found that impulsivity did not differentiate those with good vs poor outcomes at the time of treatment discharge those with higher impulsivity had poorer outcomes at 2-year follow up. Given the controlled environment of inpatient treatment, it would be helpful to know if impulsivity relates to long-term outcomes and readmission even if data do not support a relationship between impulsivity and hospital discharge outcomes.

## 5 Conclusion

This study utilized a large, primarily adult inpatient sample treated in a specialized inpatient treatment program for EDs to confirm findings from prior research showing lower impulsivity among those with AN-R compared to AN-BP and BN. This is also among the first studies to examine impulsivity in ARFID or OSFED diagnostic groups in relation to other ED diagnoses and results preliminarily suggest no significant differences for these groups, although future research with larger samples is needed. Additionally, with the exception of bulimia symptoms at admission, transdiagnostically we found that impulsivity, reward sensitivity, and punishment sensitivity were not closely related to weight, hospital course, or ED symptoms at admission or discharge. Contrary to prior research in outpatient treatment samples, self-reported levels of trait impulsivity, reward sensitivity, and punishment sensitivity were not associated with increased likelihood of treatment-interfering behaviors during hospitalization or poorer outcomes at discharge from a specialized inpatient treatment program for EDs. Impulsivity may be a key factor to consider when deciding between outpatient versus inpatient levels of care. Future research should confirm the findings from this study using a multi-method approach to measure impulsivity and BIS/BAS including self-report, collateral report from family, and behavioral task-based testing. Future studies should explore the potential effects of different types of rewards and timing of rewards within inpatient contingency management protocols to determine if response to these interventions differs by level of trait impulsivity or BIS/BAS.

## Data availability statement

The raw data supporting the conclusions of this article will be made available by the authors, without undue reservation.

## Ethics statement

The studies involving humans were approved by Johns Hopkins Institutional Review Board. The studies were conducted in accordance with the local legislation and institutional requirements. Written informed consent for participation in this study was provided by the participants’ legal guardians/next of kin.

## Author contributions

MM: Conceptualization, Formal analysis, Investigation, Methodology, Writing – original draft, Writing – review & editing, Project administration, Visualization. CS: Supervision, Writing – review & editing, Conceptualization. IV: Writing – original draft, Writing – review & editing. AG: Supervision, Writing – review & editing.
