# Supplementary material for: Impulsivity and reward and punishment sensitivity among patients admitted to a specialized inpatient eating disorder treatment program
Source: Front Psychiatry. 2024 May 20;15:1325252. doi: 10.3389/fpsyt.2024.1325252 (PMC11145411; doi:10.3389/fpsyt.2024.1325252)
Supplement: Supplementary file 1 [file Table_1.docx]

Supplementary Material

Impulsivity and Reward and Punishment Sensitivity Among Patients in Admitted to a Specialized Inpatient Eating Disorder Treatment Program

Mary K. Martinelli, Ph.D.^*^, Colleen C. Schreyer, Ph.D., Irina A. Vanzhula, Ph.D., & Angela S. Guarda, M.D.

***Correspondence:** Mary K. Martinelli, Ph.D.: mmart136@jhu.edu

# Supplementary Tables

**Supplementary Table 1.** Hierarchical regressions for EDE-Q eating, weight, and shape concerns at program admission.

| DV | Model | Step^a^ | Predictors | *β* | *t* | *R*^2^ | *F-*change | *F* |
| --- | --- | --- | --- | --- | --- | --- | --- | --- |
| **EDE-Q Eating Concern**^b^ | 1 | 1 | Age | -0.05 | -0.76 | 0.19 | **12.59*** | **12.59*** |
|  |  |  | Depression | 0.00 | 0.02 |  |  |  |
|  |  |  | Anxiety | 0.43 | **3.97*** |  |  |  |
|  |  | 2 | BIS-11 AT | -0.08 | -0.78 | 0.20 | 0.67 | **6.59*** |
|  |  |  | BIS-11 MT | 0.12 | 1.30 |  |  |  |
|  |  |  | BIS-11 NP | -0.02 | -0.17 |  |  |  |
|  | 2 | 2 | BIS-11 Total | 0.04 | 0.46 | 0.19 | 0.21 | **9.45*** |
|  | 3 | 1 | Depression | -0.01 | -0.05 | 0.18 | **18.64*** | **18.64*** |
|  |  |  | Anxiety | 0.43 | **4.04*** |  |  |  |
|  |  | 2 | BAS DR | 0.04 | 0.54 | 0.20 | 0.93 | **6.83*** |
|  |  |  | BAS FS | -0.01 | -0.07 |  |  |  |
|  |  |  | BAS RR | 0.13 | 1.42 |  |  |  |
|  |  |  | BIS subscale | -0.06 | -0.66 |  |  |  |
| **EDE-Q Weight Concern**^b^ | 1 | 1 | Age | 0.05 | 0.76 | 0.29 | **21.87*** | **21.87*** |
|  |  |  | Depression | 0.18 | 1.81 |  |  |  |
|  |  |  | Anxiety | 0.38 | **3.77*** |  |  |  |
|  |  | 2 | BIS-11 AT | -0.15 | -1.55 | 0.30 | 0.86 | **11.34*** |
|  |  |  | BIS-11 MT | 0.00 | 0.04 |  |  |  |
|  |  |  | BIS-11 NP | 0.05 | 0.52 |  |  |  |
|  | 2 | 2 | BIS-11 Total | -0.06 | -0.77 | 0.29 | 0.60 | **16.51*** |
|  | 3 | 1 | Depression | 0.19 | 1.89 | 0.28 | **32.60*** | **32.60*** |
|  |  |  | Anxiety | 0.37 | **3.73*** |  |  |  |
|  |  | 2 | BAS DR | 0.00 | 0.01 | 0.29 | 0.19 | **10.78*** |
|  |  |  | BAS FS | -0.05 | -0.65 |  |  |  |
|  |  |  | BAS RR | 0.05 | 0.61 |  |  |  |
|  |  |  | BIS subscale | -0.05 | -0.61 |  |  |  |
| **EDE-Q Shape Concern**^c^ | 1 | 1 | Age | 0.03 | 0.45 | 0.29 | **22.51*** | **22.51*** |
|  |  |  | Depression | 0.21 | 2.06* |  |  |  |
|  |  |  | Anxiety | 0.36 | **3.59*** |  |  |  |
|  |  | 2 | BIS-11 AT | -0.12 | -1.21 | 0.30 | 0.51 | **11.41*** |
|  |  |  | BIS-11 MT | 0.03 | 0.33 |  |  |  |
|  |  |  | BIS-11 NP | 0.06 | 0.60 |  |  |  |
|  | 2 | 2 | BIS-11 Total | -0.01 | -0.08 | 0.29 | 0.01 | **16.78*** |
|  | 3 | 1 | Depression | 0.21 | 2.12* | 0.29 | **33.83*** | **33.83*** |
|  |  |  | Anxiety | 0.36 | **3.58*** |  |  |  |
|  |  | 2 | BAS DR | 0.06 | 0.79 | 0.30 | 0.23 | **11.22*** |
|  |  |  | BAS FS | -0.03 | -0.37 |  |  |  |
|  |  |  | BAS RR | 0.01 | 0.08 |  |  |  |
|  |  |  | BIS subscale | 0.02 | 0.26 |  |  |  |

*Note.* Results with asterisk (*) are significant based on unadjusted *p* <.05; Bolded results with asterisk (**bold, ***) are significant based on *p <.*0167 (adjustment for multiple tests) ; EDE-Q, Eating Disorder Examination Questionnaire; *BIS-11*, Barratt Impulsiveness Scale; *AT, Attentional*; *MT*, Motor; *NP*, Non-Planning; *BAS*, Behavioral Activation Scale; *DR*, Drive; *FS*, Fun Seeking; *RR*, Reward Responsiveness; *BIS*, Behavioral Inhibition Scale; *Depression*, Beck Depression Inventory (BDI-II) total score; *Anxiety*, State-Trait Anxiety Inventory Trait subscale (STAI-T).

^a^Only variables unique to each step are presented

^b^*n* = 169

^c^*n* = 168

**Supplementary Table 2.** Hierarchical regressions for EDI-2 drive for thinness and body dissatisfaction at program admission (*N* = 228).

| DV | Model | Step^a^ | Predictors | *β* | *t* | *R*^2^ | *F-*change | *F* |
| --- | --- | --- | --- | --- | --- | --- | --- | --- |
| **EDI-2 Drive for Thinness** | 1 | 1 | Age | -0.02 | -0.37 | 0.26 | **26.34*** | **26.34*** |
|  |  |  | Depression | 0.28 | **3.26*** |  |  |  |
|  |  |  | Anxiety | 0.27 | **3.16*** |  |  |  |
|  |  | 2 | BIS-11 AT | -0.07 | -0.87 | 0.27 | 0.42 | **13.28*** |
|  |  |  | BIS-11 MT | 0.01 | 0.20 |  |  |  |
|  |  |  | BIS-11 NP | -0.03 | -0.34 |  |  |  |
|  | 2 | 2 | BIS-11 Total | -0.06 | -0.88 | 0.26 | 0.78 | **19.93*** |
|  | 3 | 1 | Depression | 0.28 | **3.25*** | 0.26 | **39.59*** | **39.59*** |
|  |  |  | Anxiety | 0.27 | **3.18*** |  |  |  |
|  |  | 2 | BAS DR | 0.05 | 0.74 | 0.27 | 0.78 | **13.65*** |
|  |  |  | BAS FS | -0.03 | -0.48 |  |  |  |
|  |  |  | BAS RR | 0.05 | 0.66 |  |  |  |
|  |  |  | BIS subscale | 0.06 | 0.84 |  |  |  |
| **EDI-2 Body Dissatisfaction** | 1 | 1 | Age | 0.02 | 0.43 | 0.32 | **35.01*** | **35.01*** |
|  |  |  | Depression | 0.34 | **4.10*** |  |  |  |
|  |  |  | Anxiety | 0.27 | **3.26*** |  |  |  |
|  |  | 2 | BIS-11 AT | -0.06 | -0.75 | 0.32 | 0.22 | **17.43*** |
|  |  |  | BIS-11 MT | 0.02 | 0.35 |  |  |  |
|  |  |  | BIS-11 NP | -0.01 | -0.11 |  |  |  |
|  | 2 | 2 | BIS-11 Total | -0.02 | -0.38 | 0.32 | 0.14 | **26.19*** |
|  | 3 | 1 | Depression | 0.34 | **4.14*** | 0.32 | **52.61*** | **52.61*** |
|  |  |  | Anxiety | 0.27 | **3.26*** |  |  |  |
|  |  | 2 | BAS DR | -0.07 | -1.10 | 0.33 | 0.50 | **17.71*** |
|  |  |  | BAS FS | -0.03 | -0.42 |  |  |  |
|  |  |  | BAS RR | 0.02 | 0.25 |  |  |  |
|  |  |  | BIS subscale | 0.02 | 0.32 |  |  |  |

*Note.* Results with asterisk (*) are significant based on unadjusted *p* <.05; Bolded results with asterisk (**bold, ***) are significant based on *p <.*0167 (adjustment for multiple tests); *EDI-2*, Eating Disorder Inventory 2; *BIS-11*, Barratt Impulsiveness Scale; *AT, Attentional*; *MT*, Motor; *NP*, Non-Planning; *BAS*, Behavioral Activation Scale; *DR*, Drive; *FS*, Fun Seeking; *RR*, Reward Responsiveness; *BIS*, Behavioral Inhibition Scale; *Depression*, Beck Depression Inventory (BDI-II) total score; *Anxiety*, State-Trait Anxiety Inventory Trait subscale (STAI-T).

^a^Only variables unique to each step are presented

**Supplementary Table 3.** Binary logistic regression models predicting treatment dropout (*N* = 228).

| Model^a^ | Predictors^b^ | *B* | Wald | Sig. | Exp*(B)* | 95% C.I. for Exp(B) | |
| --- | --- | --- | --- | --- | --- | --- | --- |
|  |  |  |  |  |  | Lower | Upper |
| 1 | Age | 0.01 | 1.75 | 0.186 | 1.01 | 0.99 | 1.04 |
|  | Depression | -0.01 | 0.50 | 0.478 | 0.99 | 0.96 | 1.02 |
|  | Anxiety | -0.00 | 0.01 | 0.905 | 0.99 | 0.97 | 1.03 |
|  | Admission BMI | -0.09 | 6.45 | **0.011*** | 0.91 | 0.85 | 0.98 |
|  | BIS-11 AT | 0.06 | 2.03 | 0.155 | 1.06 | 0.98 | 1.15 |
|  | BIS-11 MT | 0.04 | 0.96 | 0.326 | 1.04 | 0.96 | 1.12 |
|  | BIS-11 NP | -0.04 | 1.48 | 0.223 | 0.96 | 0.90 | 1.03 |
| 2 | Age | 0.01 | 1.16 | 0.282 | 1.01 | 0.99 | 1.03 |
|  | Depression | -0.01 | 0.27 | 0.602 | 0.99 | 0.96 | 1.02 |
|  | Anxiety | -0.00 | 0.00 | 0.973 | 1.00 | 0.97 | 1.03 |
|  | Admission BMI | -0.10 | 6.67 | **0.010*** | 0.91 | 0.85 | 0.98 |
|  | BIS-11 Total | 0.01 | 0.67 | 0.411 | 1.01 | 0.99 | 1.04 |
| 3 | Depression | -0.01 | 0.28 | 0.594 | 0.99 | 0.96 | 1.02 |
|  | Anxiety | 0.00 | 0.02 | 0.891 | 1.00 | 0.97 | 1.03 |
|  | Admission BMI | -0.09 | 6.46 | **0.011*** | 0.91 | 0.85 | 0.98 |
|  | BAS DR | 0.06 | 1.13 | 0.289 | 1.06 | 0.95 | 1.19 |
|  | BAS FS | -0.00 | 0.00 | 0.992 | 1.00 | 0.88 | 1.13 |
|  | BAS RR | -0.05 | 0.57 | 0.452 | 0.95 | 0.83 | 1.09 |
|  | BIS subscale | 0.01 | 0.03 | 0.869 | 1.01 | 0.92 | 1.11 |

*Note. BMI*, body mass index (kg/m^2^); *BIS-11*, Barratt Impulsiveness Scale; *AT, Attentional*; *MT*, Motor; *NP*, Non-Planning; *BAS*, Behavioral Activation Scale; *DR*, Drive; *FS*, Fun Seeking; *RR*, Reward Responsiveness; *BIS*, Behavioral Inhibition Scale; *Depression*, Beck Depression Inventory (BDI-II) total score; *Anxiety*, State-Trait Anxiety Inventory Trait subscale (STAI-T).

^a^Overall model significance for Model 1: *χ*^2^(7) = 14.22, *p* =. 047, Model 2: *χ*^2^(5) = 11.14, *p* =. 049, Model 3: *χ*^2^(7) = 10.38, *p* = .168.

^b^Results presented are from full model with all predictors and covariates included

**p<.*0167 (adjusted for multiple comparisons)

**Supplementary Table 4.** Binary logistic regression models predicting whether patients exhibited problematic behavior during admission (*N* = 228).

| Model^a^ | Predictors^b^ | *B* | Wald | Sig. | Exp*(B)* | 95% C.I. for Exp(B) | |
| --- | --- | --- | --- | --- | --- | --- | --- |
|  |  |  |  |  |  | Lower | Upper |
| 1 | Age | -0.03 | 6.14 | 0.013 | 0.97 | 0.94 | 0.99 |
|  | Depression | 0.02 | 1.07 | 0.301 | 1.02 | 0.98 | 1.06 |
|  | Anxiety | 0.02 | 1.75 | 0.186 | 1.02 | 0.99 | 1.06 |
|  | BIS-11 AT | 0.04 | 0.93 | 0.335 | 1.04 | 0.96 | 1.14 |
|  | BIS-11 MT | -0.03 | 0.45 | 0.504 | 0.97 | 0.90 | 1.05 |
|  | BIS-11 NP | 0.01 | 0.14 | 0.706 | 1.01 | 0.95 | 1.08 |
| 2 | Age | -0.04 | 7.62 | **0.006*** | 0.97 | 0.94 | 0.99 |
|  | Depression | 0.02 | 1.14 | 0.286 | 1.02 | 0.98 | 1.06 |
|  | Anxiety | 0.03 | 2.97 | 0.085 | 1.03 | 1.00 | 1.07 |
|  | BIS-11 Total | 0.01 | 0.28 | 0.599 | 1.01 | 0.98 | 1.03 |
| 3 | Depression | 0.02 | 0.85 | 0.356 | 1.02 | 0.98 | 1.05 |
|  | Anxiety | 0.04 | 4.78 | 0.029 | 1.04 | 1.00 | 1.08 |
|  | BAS DR | -0.03 | 0.22 | 0.639 | 0.97 | 0.86 | 1.10 |
|  | BAS FS | 0.07 | 1.14 | 0.285 | 1.07 | 0.94 | 1.22 |
|  | BAS RR | 0.00 | 0.00 | 0.998 | 1.00 | 0.87 | 1.15 |
|  | BIS subscale | -0.03 | 0.38 | 0.537 | 0.97 | 0.88 | 1.07 |

*Note. BIS-11*, Barratt Impulsiveness Scale; *AT, Attentional*; *MT*, Motor; *NP*, Non-Planning; *BAS*, Behavioral Activation Scale; *DR*, Drive; *FS*, Fun Seeking; *RR*, Reward Responsiveness; *BIS*, Behavioral Inhibition Scale; *Depression*, Beck Depression Inventory (BDI-II) total score; *Anxiety*, State-Trait Anxiety Inventory Trait subscale (STAI-T).

^a^Overall model significance for Model 1: *χ*^2^(6) = 25.98, *p* <.001, Model 2: *χ*^2^(4) = 24.84, *p* <.001, Model 3: *χ*^2^(6) = 18.06, *p* = .001.

^b^Results presented are from full model with all predictors and covariates included

**p<.*0167 (adjusted for multiple comparisons)

**Supplementary Table 5.** Hierarchical regressions for T2 EDI-2 bulimia symptoms (*n* = 72).

| Model | Step^a^ | Predictors | *β* | *t* | *R*^2^ | *F-*change | *F* |
| --- | --- | --- | --- | --- | --- | --- | --- |
|  | 1 | Age | -0.09 | -1.06 | 0.57 | **22.27*** | **22.27*** |
|  |  | Depression | -0.13 | -1.03 |  |  |  |
|  |  | Anxiety | 0.18 | 1.39 |  |  |  |
|  |  | T1 EDI-2 Bulimia | 0.73 | **9.00*** |  |  |  |
| 1 | 2 | BIS-11 AT | 0.06 | 0.57 | 0.62 | 2.51 | **14.66*** |
|  |  | BIS-11 MT | 0.20 | 2.05***** |  |  |  |
|  |  | BIS-11 NP | 0.00 | 0.05 |  |  |  |
| 2 | 2 | BIS-11 Total | 0.22 | 2.45***** | 0.61 | 6.02***** | **20.35*** |
|  | 1 | Depression | -0.14 | -1.04 | 0.57 | **29.26*** | **29.26*** |
|  |  | Anxiety | 0.17 | 1.32 |  |  |  |
|  |  | T1 EDI-2 Bulimia | 0.73 | **9.06*** |  |  |  |
| 3 | 2 | BAS DR | -0.06 | -0.69 | 0.63 | 2.68***** | **15.31*** |
|  |  | BAS FS | 0.10 | 1.11 |  |  |  |
|  |  | BAS RR | -0.24 | -2.28***** |  |  |  |
|  |  | BIS subscale | -0.02 | -0.23 |  |  |  |

*Note.* Results with asterisk (*) are significant based on unadjusted *p* <.05; Bolded results with asterisk (**bold**, *) are significant based on *p <.*0167 (adjustment for multiple tests); *EDI-2*, Eating Disorder Inventory 2; *T1*, time 1 (admission); *T2*, time 2 (discharge); *BIS-11*, Barratt Impulsiveness Scale; *AT, Attentional*; *MT*, Motor; *NP*, Non-Planning; *BAS*, Behavioral Activation Scale; *DR*, Drive; *FS*, Fun Seeking; *RR*, Reward Responsiveness; *BIS*, Behavioral Inhibition Scale *Depression*, total Beck Depression Inventory (BDI-II) score; *Anxiety*, State-Trait Anxiety Inventory trait subscale (STAI-T).

^a^Only variables unique to each step are presented

**Supplementary Table 6.** Hierarchical regressions for T2 EDE-Q eating, weight, and shape concerns.

| DV | Model | Step^a^ | Predictors | *β* | *t* | *R*^2^ | *F-*change | *F* |
| --- | --- | --- | --- | --- | --- | --- | --- | --- |
| **T2 EDE-Q Eating Concern**^b^ | 1 | 1 | Age | -0.06 | -0.81 | 0.29 | **13.88*** | **13.88*** |
|  |  |  | Depression | -0.14 | -1.34 |  |  |  |
|  |  |  | Anxiety | 0.42 | **3.78*** |  |  |  |
|  |  |  | T1 Eating Concern | 0.32 | **4.07*** |  |  |  |
|  |  | 2 | BIS-11 AT | -0.15 | -1.43 | 0.31 | 1.20 | **8.48*** |
|  |  |  | BIS-11 MT | 0.08 | 0.82 |  |  |  |
|  |  |  | BIS-11 NP | -0.07 | -0.67 |  |  |  |
|  | 2 | 2 | BIS-11 Total | -0.09 | -1.52 | 0.30 | 1.33 | **11.39*** |
|  | 3 | 1 | Depression | -0.15 | -1.40 | 0.29 | **18.33*** | **18.33*** |
|  |  |  | Anxiety | 0.42 | **3.78*** |  |  |  |
|  |  |  | T1 Eating Concern | 0.33 | **4.17*** |  |  |  |
|  |  | 2 | BAS DR | -0.08 | -0.96 | 0.31 | 0.75 | **8.22*** |
|  |  |  | BAS FS | -0.08 | -0.89 |  |  |  |
|  |  |  | BAS RR | 0.02 | 0.25 |  |  |  |
|  |  |  | BIS subscale | 0.05 | 0.54 |  |  |  |
| **T2 EDE-Q Weight Concern**^c^ | 1 | 1 | Age | -0.05 | -0.88 | 0.53 | **37.59*** | **37.59*** |
|  |  |  | Depression | 0.00 | -0.04 |  |  |  |
|  |  |  | Anxiety | 0.20 | 2.13* |  |  |  |
|  |  |  | T1 Weight Concern | 0.62 | **9.11*** |  |  |  |
|  |  | 2 | BIS-11 AT | -0.12 | -1.34 | 0.54 | 0.80 | **21.73*** |
|  |  |  | BIS-11 MT | 0.07 | 0.89 |  |  |  |
|  |  |  | BIS-11 NP | -0.02 | -0.19 |  |  |  |
|  | 2 | 2 | BIS-11 Total | -0.03 | -0.43 | 0.53 | 0.18 | **29.92*** |
|  | 3 | 1 | Depression | -0.01 | -0.10 | 0.53 | **49.94*** | **49.94*** |
|  |  |  | Anxiety | 0.20 | 2.18* |  |  |  |
|  |  |  | T1 Weight Concern | 0.62 | **9.08*** |  |  |  |
|  |  | 2 | BAS DR | 0.04 | 0.56 | 0.53 | 0.10 | **20.88*** |
|  |  |  | BAS FS | -0.02 | -0.26 |  |  |  |
|  |  |  | BAS RR | -0.03 | -0.36 |  |  |  |
|  |  |  | BIS subscale | 0.02 | 0.21 |  |  |  |
| **T2 EDE-Q Shape Concern**^c^ | 1 | 1 | Age | -0.05 | -0.75 | 0.50 | **32.94*** | **32.94*** |
|  |  |  | Depression | -0.08 | -0.81 |  |  |  |
|  |  |  | Anxiety | 0.28 | **2.95*** |  |  |  |
|  |  |  | T1 Shape Concern | 0.58 | **8.19*** |  |  |  |
|  |  | 2 | BIS-11 AT | -0.20 | -2.23* | 0.53 | 2.28 | **20.35*** |
|  |  |  | BIS-11 MT | -0.02 | -0.22 |  |  |  |
|  |  |  | BIS-11 NP | 0.02 | 0.18 |  |  |  |
|  | 2 | 2 | BIS-11 Total | -0.13 | -1.92 | 0.51 | 3.68 | **27.62*** |
|  | 3 | 1 | Depression | -0.08 | -0.85 | 0.50 | **43.88*** | **43.88*** |
|  |  |  | Anxiety | 0.28 | **2.98*** |  |  |  |
|  |  |  | T1 Shape Concern | 0.58 | **8.19*** |  |  |  |
|  |  | 2 | BAS DR | -0.02 | -0.21 | 0.50 | 0.16 | **18.42*** |
|  |  |  | BAS FS | -0.01 | -0.08 |  |  |  |
|  |  |  | BAS RR | -0.03 | -0.33 |  |  |  |
|  |  |  | BIS subscale | 0.06 | 0.68 |  |  |  |

*Note.* Results with asterisk (*) are significant based on unadjusted *p* <.05; Bolded results with asterisk (**bold, ***) are significant based on *p <.*0167 (adjustment for multiple tests); EDE-Q, Eating Disorder Examination Questionnaire; *T1*, time 1 (admission); *T2*, time 2 (discharge); *BIS-11*, Barratt Impulsiveness Scale; *AT, Attentional*; *MT*, Motor; *NP*, Non-Planning; *BAS*, Behavioral Activation Scale; *DR*, Drive; *FS*, Fun Seeking; *RR*, Reward Responsiveness; *BIS*, Behavioral Inhibition Scale; *Depression*, Beck Depression Inventory (BDI-II) total score; *Anxiety*, State-Trait Anxiety Inventory Trait subscale (STAI-T).

^a^Only variables unique to each step are presented

^b^*n* = 138

^c^*n* = 137
